# Enhancing Document Classification Through Multimodal Image-Text Classification: Insights from Fine-Tuned CLIP and Multimodal Deep Fusion

**DOI:** 10.3390/s25247596

**Published:** 2025-12-15

**Authors:** Hosam Aljuhani, Mohamed Yehia Dahab, Yousef Alsenani

**Affiliations:** 1 Department of Computer Science, Faculty of Computing and Information Technology, King Abdulaziz University, Jeddah 22254, Saudi Arabia; mdahab@kau.edu.sa; 2 Department of Information Systems, Faculty of Computing and Information Technology, Center of Research Excellence in Artificial Intelligence and Data Science, King Abdulaziz University, Jeddah 22254, Saudi Arabia; yalsenani@kau.edu.sa

**Keywords:** multimodal deep learning, medical diagnosis, vision-language models, CLIP, fine-tuning, transfer learning, hybrid fusion, medical image classification

## Abstract

Foundation models excel on general benchmarks but often underperform in clinical settings due to domain shift between internet-scale pretraining data and medical data. Multimodal deep learning, which jointly leverages medical images and clinical text, is promising for diagnosis, yet it remains unclear whether domain adaptation is better achieved by fine-tuning large vision–language models or by training lighter, task-specific architectures. We address this question by introducing PairDx, a balanced dataset of 22,665 image–caption pairs spanning six medical document classes, curated to reduce class imbalance and support fair, reproducible comparisons. Using PairDx, we develop and evaluate two approaches: (i) PairDxCLIP, a fine-tuned CLIP (ViT-B/32), and (ii) PairDxFusion, a custom hybrid model that combines ResNet-18 visual features and GloVe text embeddings with attention-based fusion. Both adapted models substantially outperform a zero-shot CLIP baseline (61.18% accuracy) and a specialized model, BiomedCLIP, which serves as an additional baseline and achieves 66.3% accuracy. Our fine-tuned CLIP (PairDxCLIP) attains 93% accuracy and our custom fusion model (PairDxFusion) reaches 94% accuracy on a held-out test set. Notably, PairDxFusion achieves this high accuracy with 17 min, 55 s of training time, nearly four times faster than PairDxCLIP (65 min, 52 s), highlighting a practical efficiency–performance trade-off for clinical deployment. The testing time also outperforms the specialized model—BiomedCLIP (0.387 s/image). Our results demonstrate that carefully constructed domain-specific datasets and lightweight multimodal fusion can close the domain gap while reducing computational cost in healthcare decision support.

## 1. Introduction

The integration of multimodal deep learning and artificial intelligence (AI) is transforming medical diagnostics by enabling new forms of data integration and automated clinical support [1]. The significance of this paradigm shift is highlighted by the projection that the global AI in the healthcare market will surpass $188 billion by 2030, with an estimated annual growth of 37% [2]. In current clinical workflows, decision-making almost always requires synthesizing information from multiple sources: up to 80% of medical records include both images and associated textual reports, and approximately 62% of diagnostic errors have been linked to failures in integrating multimodal data [3,4]. Studies show that leveraging both clinical text and medical images results in measurable improvements, with multimodal AI systems delivering an average increase of 6.2 percentage points in diagnostic accuracy over unimodal models, as documented in systematic reviews [3,5]. Therefore, building clinically useful tools increasingly depends on deploying advanced AI models that can ingest, process, and meaningfully combine heterogeneous medical data [6].

Importantly, the specific task addressed in this work is not direct disease or diagnosis prediction, but rather medical document classification at the modality level, that is, assigning a given image-caption pair to one of six medical document classes, such as radiology, endoscopy, ECG, and others. This distinction has impactful clinical implications: while disease prediction targets patient-specific outcomes, modality-level document classification is foundational to organizing large-scale medical databases, powering automated clinical triage, and enabling downstream AI workflows such as evidence retrieval and clinical cohort identification. Accurate automated classification at the modality level accelerates the structuring of raw clinical data, reducing manual overhead and supporting both research and quality improvement initiatives without requiring disease-level annotation or risking overinterpretation of AI outputs in patient management contexts.

The advent of large, pre-trained vision-language models (VLMs), such as Contrastive Language-Image Pre-training (CLIP), marks a major milestone in learning generalizable representations from hundreds of millions of image-text pairs [7,8,9,10,11]. While CLIP’s zero-shot capabilities have set strong baselines in generic vision-language benchmarks, their impact and optimal use in medical settings—especially for clinical document modality classification—remains to be systematically assessed. In this paper, we rigorously evaluate foundation models like CLIP on a multi-class, real-world modality classification task, explicitly focused on distinguishing among medical document types rather than inferring disease or diagnosis. We conduct a systematic comparison using a newly curated, balanced multimodal dataset spanning a range of clinically relevant imaging modalities. Our empirical analyses compare the zero-shot CLIP model, a variant fine-tuned on our domain-specific data (PairDxCLIP), a custom-designed deep learning architecture (PairDxFusion), and a specialized biomedical vision-language model (BiomedCLIP [12]). These results provide new insight into the strengths and limitations of leading approaches and clarify the practical value of modality-level document classification for clinical informatics and medical AI infrastructure.

### 1.1. Background and Motivation

Recognizing the well-documented shortcomings of unimodal AI is integral to motivating this work. Unimodal models, although successful within narrow domains, remain fundamentally limited when exposed to the complexity of real-world clinical environments; for example, single-modality models can experience error rates as high as 30% when deployed outside the controlled settings in which they are developed [1,13]. Multimodal learning directly addresses these limitations by fusing multiple complementary information streams, such as imaging and free-text notes, these methods have demonstrated substantial improvements in diagnostic sensitivity and specificity [3,6]. The specialized models like BiomedCLIP, have demonstrated substantial improvements in diagnostic sensitivity and specificity [14]. But still they need additional training on the domain-specific, more structured data and pre-processing to achieve the best performance.

Despite these advances, important questions linger about the performance of modern multimodal models in healthcare. For example, the cross-domain effectiveness of foundation models like CLIP remains unclear, while CLIP achieves near state-of-the-art results (often 75–85% accuracy) in general vision-language tasks, its zero-shot accuracy on challenging medical datasets can drop to the range of 50–65% [15]. This gap highlights the domain adaptation problem. A performance comparison between customized architectures and optimized foundation models is critical, particularly since multimodal datasets in medicine are often imbalanced or lack sufficient representation of all classes, further complicating robust benchmarking [16,17].

Foundation models such as CLIP have achieved significant milestones in other fields due to their pre-training on gigantic web-scale datasets (e.g., CLIP is pre-trained on 400 million image-text pairs [7]). However, transferring these capabilities to healthcare is not direct, as the specialized nature of medical images and the prevalence of technical language, negations, and fine-grained features in medical text poses unique challenges [9,18]. For example, over 40% of medical image-text pairs can include non-standard abbreviations or domain-specific terminology, making generic representations less effective without adaptation [8].

To address such gaps, research has evolved along two principal paths. The first involves developing novel, custom deep learning models from scratch, leveraging methods like GloVe embeddings for textual data and CNNs for images, integrated using early, hybrid, or late fusion mechanisms [3,19,20]. The second, more recent path focuses on adapting large pre-trained models such as CLIP to domain-specific datasets via fine-tuning or parameter-efficient adaptation [9,10,21]. Notably, this process remains bottlenecked by the scarcity of labeled medical images. Against this backdrop, our study is driven by the need to empirically characterize the effectiveness of both strategies, using a diverse and balanced multi-category medical dataset as a benchmark.

### 1.2. Impact of Research

The impact of this research is multifaceted, with considerable implications for both the academic and clinical domains in medical AI. Academically, the development and public release of the PairDx dataset, comprising 22,665 image–caption pairs across six medical classes, creates a valuable benchmark for future studies. This is particularly notable as recent analyses reveal that over 70% of existing medical AI studies use imbalanced or single-class datasets [22], reducing their real-world utility. By establishing a standardized, balanced resource, our work facilitates the reproducible evaluation of vision-language models in realistic medical contexts.

From a practical perspective, the growing need to manage ever-increasing volumes of medical data underscores this research. It is reported that worldwide, hospitals generate approximately 50 petabytes (PB) of imaging data per year, and digitalization efforts are only accelerating this trend [23]. Deploying reliable automated models eases this data burden: for instance, automated classification and triage of radiology images has cut average diagnosis times by 20–30% in pilot studies [24,25]. Our results demonstrate that the fine-tuned PairDxCLIP model achieves 93% accuracy, while our custom PairDxFusion attains 94% accuracy on the held-out test set. The specialized model BiomedCLIP, included as a second baseline, achieves 66.3% accuracy. These findings show that tailored fine-tuning and efficient hybrid modeling can both meet clinical accuracy thresholds and improve deployment speed, with PairDxFusion training nearly four times faster than PairDxCLIP.

Additionally, the architectural and empirical insights gained from our comparative analysis can guide future model development, informing choices about pre-training, adaptation, and data requirements. The broader foray into multimodal learning has the potential for direct patient impact, improved early detection (e.g., reducing missed diagnoses in rural clinics, which are reported at up to 15% nationally [26]) and streamlined workflows for overextended clinicians. Thus, our research not only advances the multimodal learning discourse but also lays groundwork for next-generation diagnostic tools that align with both clinical needs and the realities of modern healthcare systems.

### 1.3. Challenges in Multimodal Medical AI

Integrating multimodal AI into medical diagnostics is fraught with technical, ethical, and operational challenges. Technically, the heterogeneity of medical data is substantial. Modern hospitals may use over 100 unique imaging and data formats, and as much as 80% of clinical data exists in unstructured or semi-structured forms [6,27]. Images can vary widely in resolution and modality (CT, MRI, X-ray, etc.), while textual data ranges from informal clinical notes to formal EHRs, complicating preprocessing and harmonization [18,19].

Another persistent obstacle is the scarcity of labeled medical data. Estimates suggest that fewer than 5% of clinical images are annotated for AI training purposes globally [13]. Privacy regulations (such as HIPAA) and ethical guidelines further restrict the distribution and use of such data, necessitating data-efficient learning techniques, like transfer learning, semi-supervised learning, or federated learning to maximize the utility of available labeled samples [21,28].

Ethical issues remain paramount in clinical AI deployment. Interpretability is essential for trust. One systematic review found that less than 10% of published medical AI models include provisions for meaningful explanation of their predictions [29]. Bias, often rooted in imbalanced datasets, contributes to unequal performance across demographic groups, which can exacerbate existing healthcare disparities [5,30]. Addressing these issues demands collaboration between clinicians, data scientists, and ethicists to develop transparent and fair models [31].

Finally, there are substantial operational hurdles to clinical integration. For example, many healthcare providers cite insufficient training as a barrier to AI adoption [32], while complex user interfaces or poor EHR interoperability can slow or prevent clinical uptake [33]. Ensuring reliability and resilience in real-world settings is critical for realizing sustained improvements in patient care, requiring not only technical innovation but also user-centered system design [6].

### 1.4. Problem Statement

Despite rapid progress, there remain a number of unresolved issues hindering the application of advanced multimodal models to medical document classification [34,35]. For example, while models like CLIP achieve over 75% accuracy in general settings, their baseline zero-shot performance on specialized, multi-class medical datasets (spanning radiology, endoscopy, ECG, and charts) can dip as low as 50–61%, as observed in benchmarks [15]. Quantifying this gap is essential for understanding the true limitations of foundational models in clinical applications.

Moreover, the field lacks comprehensive comparisons between extensively fine-tuned foundation models and custom purpose-built multimodal models within the same, balanced medical context. Without such head-to-head evaluations, it is impossible to know whether incremental improvements in foundation models justify their resource needs or whether lighter, task-specific architectures can suffice. The lack of high-quality, balanced datasets exacerbates these questions—analyses of public medical datasets reveal class imbalance and as few as several dozen samples per minority class [22,36]. Addressing these gaps necessitates new, well-curated testbeds for benchmarking and empirical studies that directly contrast prevailing methods on common ground.

### 1.5. Research Questions and Contributions

This study is designed to address the aforementioned gaps by investigating the following research questions: RQ1: What is the baseline zero-shot classification performance of a pre-trained, general-domain CLIP model on a novel, balanced, and diverse multi-category medical image-text dataset? RQ2: How does a traditional, custom-built multimodal deep learning model using a late-fusion architecture perform on the same classification task? RQ3: To what extent can fine-tuning the pre-trained CLIP model on the target dataset improve its performance, and how does this adapted model compare to both the zero-shot baseline and the custom-built deep learning model?

This work provides the following key contributions in response to our research questions. First, we present “PairDx”, a new, balanced multimodal benchmark for medical image-text classification, curated from the MultiCaRe dataset and consisting of 22,665 image–caption pairs across six medical categories; this dataset will be made publicly available. To quantify the baseline domain gap, we evaluate the zero-shot performance of a pre-trained CLIP model on PairDx, reporting an initial accuracy of 61.18%. We also benchmark BiomedCLIP, a specialized biomedical model serving as a second baseline, which achieves 66.3% accuracy. As a strong conventional baseline, we introduce “PairDxFusion,” a custom late-fusion deep learning architecture that combines a ResNet-based image encoder with GloVe-based text representation, attaining 94% accuracy. In parallel, we show that fine-tuning CLIP on *PairDx* (“PairDxCLIP”) elevates its accuracy to 93%, effectively closing the domain gap and delivering a 32 percentage point gain over its zero-shot baseline, underscoring the transformative potential of domain adaptation in medical multimodal classification. Also, we show that the custom model—PairDxFusion achieves 94% accuracy with 17 min, 55 s training time, nearly four times faster than PairDxCLIP (65 min, 52 s), highlighting a practical efficiency–performance trade-off for clinical deployment. The testing time also outperforms the specialized model—BiomedCLIP (0.387 s/image).

This paper is structured as follows: Section 2 reviews related work in multimodal medical AI and domain adaptation; Section 3 details the PairDx dataset and experimental design; Section 4 presents results and analysis; Section 5 discusses implications and limitations; and Section 6 concludes with directions for future research.

### 1.6. Dataset Accessibility

The PairDx dataset is openly accessible for research under the Creative Commons Attribution 4.0 International (CC BY 4.0) license. It can be accessed and downloaded via the persistent identifier https://doi.org/10.5281/zenodo.17452236. The dataset archive follows a clear file structure: The root directory includes a metadata file (image_metadata.csv) providing the unique identifier, modality, image filename, caption, and data split for each entry. Images are organized in an images/subdirectory and are named by their unique identifiers.

For more information and detailed access instructions, please see the PairDx dataset Zenodo webpage. The Data Availability statement in this manuscript also refers to this resource.

## 2. Literature Review

Artificial intelligence (AI) has substantially transformed medical diagnostics, evolving from unimodal models focused solely on single data sources such as images or structured patient data to sophisticated multimodal architectures capable of simultaneously interpreting images, clinical text, and other modalities. This literature review critically assesses the progression of medical AI with a particular emphasis on multimodal learning, systematically compares existing works, and identifies current limitations and research gaps directly relevant to this study.

### 2.1. From Unimodal to Multimodal AI: Evolution and Justification

Early work in computer-aided diagnosis relied heavily on unimodal techniques, such as CNNs for image-based classification or NLP on free-text reports. These approaches demonstrated strong results in circumscribed tasks, such as diabetic retinopathy detection from fundus images [37] or automated ECG interpretation. However, real-world clinical settings typically require synthesizing multiple, diverse information streams to reach an accurate diagnosis [19,31]. Unimodal models often fail to generalize due to their limited context, with documented drops in robustness and accuracy outside curated settings [31].

Multimodal Machine Learning (MML) emerged to tackle these shortcomings, enabling joint modeling of images, text, and structured data. Empirical evidence shows significant benefits: Longhi et al. [3] report a mean improvement of 6.2 percentage points in AUC using multimodal over unimodal models, and a systematic review found that 91% of examined multimodal approaches outperformed their single-modality counterparts [5]. For instance, Ye et al. [38] demonstrated that combining structured and unstructured data (free text) increased the accuracy of injury prediction models from 50.08% (structured-only) to 75.00% (hybrid). These findings confirm the tangible value of multimodal integration in clinical AI.

### 2.2. Review and Comparison of Key Multimodal Studies

To clarify the current state of research, we present a comparative summary of representative studies in multimodal medical AI. Table 1 highlights core features, participant or dataset characteristics, intervention/model type, primary outcomes, and key limitations based on the cross-verified literature:

These representative works collectively show that the field is trending towards larger datasets, deeper fusion strategies (e.g., transformers, graph neural nets), and domain-adapted models. Performance improvements are consistently reported, particularly when images and free-text are integrated. However, external real-world validation, generalizability, and clinical deployment remain insufficiently addressed.

### 2.3. Vision-Language Models and Medical Domain Adaptation

The introduction of foundation models like CLIP marks a major technological advance due to their large-scale vision-language pretraining [7]. Direct use of CLIP in medicine is hampered by domain shifts, medical images and clinical text differ substantially from internet data [9,18]. Two dominant adaptation approaches have emerged: Domain-Specific Pre-training and Parameter-Efficient Fine-Tuning (PEFT). Domain-Specific Pre-training: BiomedCLIP, trained on 15M PubMed Central image–caption pairs, improved accuracy on RadVQA to 80% compared to 71% for general CLIP. Iteven surpassed radiology-specialized models (BioViL) for some tasks [14,39]. Parameter-Efficient Fine-Tuning (PEFT): Instead of retraining the full model, methods like CLIPath and UniCrossAdapter introduce small, trainable adapters or cross-modal attention modules. CLIPath improved classification accuracy on PCam by 19% with minimal labeled data and short adaptation time. UniCrossAdapter achieved state-of-the-art results on report generation tasks [9,41].

These strategies validate both the strengths and limitations of current approaches: effective domain adaptation is achievable, but requires either considerable in-domain data or careful methodological choices to avoid loss of general knowledge (“catastrophic forgetting”).

### 2.4. Comparative Analysis of Landmark Fusion Models

Recent multimodal models incorporate various fusion and adaptation mechanisms. Table 2 (below) summarizes several seminal works, focusing on model architecture, dataset, outcome metrics, and notable observations.

Table 2 summarizes recent multimodal fusion models for medical diagnosis, highlighting key datasets, results, and limitations. These models (e.g., MDFormer, MedCLIP) show notable performance gains, but common issues include limited validation and generalizability. Overall, advances are clear, but further work is needed for clinical robustness and deployment.

### 2.5. Summary of Achievements and Persistent Gaps

Major advances: There is consensus that multimodal fusion yields tangible metrics gains over unimodal methods for key tasks (classification, report generation). Adaptation of large pre-trained vision-language models delivers clear performance benefits if sufficient domain knowledge is injected via fine-tuning or in-domain pretraining.

Limitations: The limitations are as follows: Robustness and Generalizability: Most works lack prospective, external, or real-world clinical validation. Performance is often sensitive to input corruptions or missing modalities [18]. Interpretability: Medical VLMs and fusion models remain “black boxes”, which limits clinical trust and adoption [1]. Fairness and Demographics: Few studies systematically address bias or evaluate models across diverse patient populations [30]. Practical Deployment: Clinical deployment faces hurdles from EHR integration, usability, and data privacy constraints.

In summary, the state-of-the-art demonstrates that multimodal and adapted VLMs hold substantial promise for medical AI, with numerous studies reporting significant increases in accuracy and robustness compared to unimodal approaches. However, there remains a need for (a) rigorously standardized, balanced, and diverse datasets; (b) systematic, fair benchmarking across methods; and (c) robust validation in real clinical scenarios.

Our present study addresses these gaps by publicly releasing a balanced dataset (PairDx) and directly comparing a zero-shot CLIP baseline, an adapted CLIP, and a custom late-fusion multimodal model under identical settings—controlled for data quality and class balance. By situating our contributions within the prior literature, we clarify both the incremental performance gains of each approach and the remaining barriers to true clinical adoption.

## 3. Proposed Methodology

**Figure 1:**
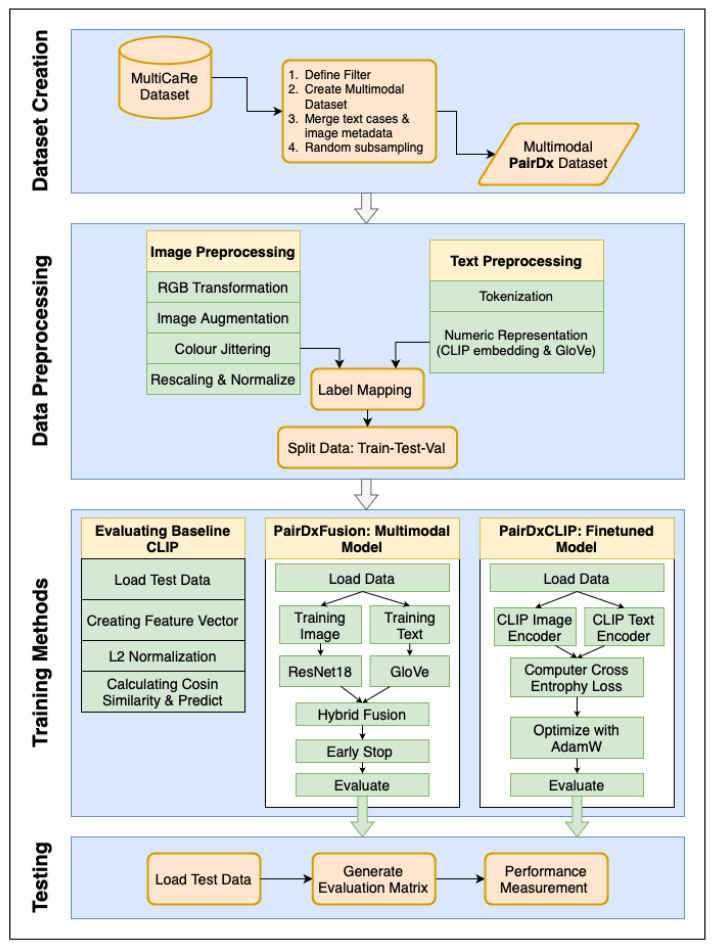
The overall workflow for our methodology. **Sky boxes** indicate steps shared across all models: (1) Creation of the balanced **PairDx** multimodal dataset, (2) Train/validation/test splitting, and (3) Standardized data preprocessing. **Inside boxes** represent steps performed separately for each model: (4) Model initialization, (5) Model-specific training, and (6) Performance evaluation. Bold arrows and enlarged text improve traceability of each stage. This explicit separation ensures fair and reproducible benchmarking.

The methodology for this study is structured to enable a systematic comparison of multimodal learning strategies in the context of medical image classification. The experimental workflow, as depicted in Figure 1, is divided into two main segments: steps shared across all model variants and procedures specific to each individual approach. The shared steps include initial dataset preparation, splitting, and preprocessing, establishing a uniform foundation for each experiment. In the model-specific portion, tasks for initializing, training (when applicable), and evaluating each method are carried out according to the unique requirements of the particular architecture under assessment. This separation ensures that every model is evaluated under equivalent conditions, supporting robust and fair benchmarking across different multimodal learning paradigms.

Shared Steps: The shared steps are as follows: Creation of the PairDx Dataset: The process begins with the aggregation and ingestion of raw, multimodal medical data from the source (MultiCaRe). Rigorous quality checks, filtering, de-duplication, and stratified sampling are employed to construct a class-balanced dataset, PairDx, including images and their paired clinical text. This step ensures that the final dataset is not only diverse but also free from major sources of bias or imbalance. Train/Validation/Test Splitting: The curated dataset is then partitioned into non-overlapping train, validation, and test sets. This segregation is conducted carefully to prevent information leakage across splits and to reflect real-world scenario generalization. Standardized Preprocessing: All images and text undergo uniform preprocessing. For images, this typically includes resizing, normalization, and modality-specific preparation. For text, standardized tokenization, cleaning, and length constraints are applied. By standardizing these steps across all subsequent modeling runs, we ensure consistency and comparability between results.

Model-Specific Steps: The model-specific steps are follows: Model Initialization: Each experimental branch initializes a specific model configuration, such as (1) a zero-shot CLIP, (2) a fine-tuned CLIP, or (3) a custom late-fusion multimodal architecture. Model hyperparameters, weights, and tokenizer/vocabulary are set up here. Model-Specific Preprocessing: Some architectures require additional processing—for example, adapting image resolution or text length, or encoding modality metadata–before training or inference. Training and Validation (If Applicable): If the model is trainable, it undergoes supervised training on the training split, with validation data used for early stopping, hyperparameter tuning, and performance monitoring. Zero-shot models bypass this, moving directly to evaluation. Evaluation: After setup (and training when required), each model—whether zero-shot or fine-tuned—is evaluated on the held-out test set. Evaluation follows identical protocols across all models, capturing metrics such as accuracy, F1, and per-class statistics to support robust benchmarking.

The depicted workflow enforces strict separation between shared operations and model-specific processing. This design maintains experimental rigor, allowing a direct, fair comparison of different multimodal strategies. Clear visual cues in Figure 1 correspond to the methodological structure outlined above. Furthermore, the bold arrows and block labels within the diagram emphasize the logical flow: from data preparation (ingestion, balancing, splitting) through to model-specific execution (initialization, training, and evaluation). This comprehensive process guarantees that every model is assessed on the same grounds, with all results traceable by stage, thereby supporting both reproducibility and clear interpretation of benchmarking outcomes. In addition, the overall workflow is shown in Algorithm 1.
**Algorithm 1** Overall Experimental Workflow.1:**function** M_AIN_W_ORKFLOW_(dataset_path, model_configs)2:    MultiCaRe←LoadRawData(dataset_path)3:    FilteredData←ApplyFilters(MultiCaRe)         ▹ Filter by age and modality4:    PairDx←CreateBalancedDataset(FilteredData)         ▹ Merge and subsample5:    train_set, val_set, test_set←LoadPredefinedSplits(PairDx)    ▹ Load 70/15/15 splits6:    results←{}7:    **for** each config in model_configs **do**8:        model←InitializeModel(config)9:        preprocessed_test_set←Preprocess(test_set, config)10:        **if** config.is_trainable **then**11:           preprocessed_train_set←Preprocess(train_set, config)12:           preprocessed_val_set←Preprocess(val_set, config)13:           trained_model←TrainModel(model, preprocessed_train_set, preprocessed_val_set)14:           results[config.name]←EvaluateModel(trained_model, preprocessed_test_set)15:        **else**16:           results[config.name]←EvaluateModel(model, preprocessed_test_set)    ▹ For zero-shot17:        **end if**18:    **end for**19:    **return** results20:**end function**

### 3.1. Dataset Curation: The PairDx Dataset

The PairDx dataset, a carefully selected, multimodal, and class-balanced collection of medical images and their textual captions, serves as the basis for this study. Through a multi-step refinement process, the dataset was taken from the extensive MultiCaRe dataset [43]. Table 3 provides a detailed breakdown of the number of image-text pairs by modality in the PairDx dataset.

We began by loading the comprehensive MultiCaRe dataset. To ensure clinical relevance and consistency, we filtered for patients at least 18 years old and selected images from 11 key medical modalities (radiology, endoscopy, medical photographs, ophthalmic imaging, electrography, chart images, pathology, ultrasound, nuclear medicine, dermatology, surgical images). After filtering, we curated an initial cohort of 103,384 images and merged these with case data and image metadata. All images were screened for personally identifiable information (PII), such as names or facial features, with masks or cropping applied as needed, and metadata fields containing PII were removed. Captions underwent an automated filter to exclude common identifiers (e.g., names, dates, IDs), followed by manual review of a subset.

To address class imbalance, we performed random subsampling, yielding a balanced PairDx dataset of 22,665 unique image-caption pairs, and created stratified splits for training (70%), validation (15%), and testing (15%). While comprehensive de-identification steps were taken on top of using an open dataset, small residual risks of re-identification remain due to rare clinical scenarios or potential linkage attacks. We therefore emphasize the importance of ethical best practices and appropriate data safeguards for any downstream use.

**Figure 2:**
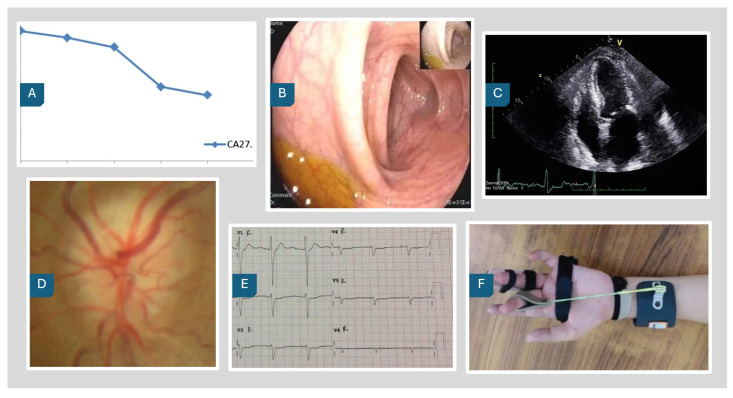
PairDx Dataset Example Images. (**A**) Chart; (**B**) Endoscopy; (**C**) Radiology; (**D**) Ophthalmic Imaging; (**E**) Electrography; (**F**) Medical Photograph.

The PairDx dataset is visually represented in Figure 2, which showcases example images from each class. These images provide a glimpse into the diverse types of images included in the dataset, such as medical photographs, endoscopy, electrography, ophthalmic imaging, radiology, and charts. Accompanying these images, Table 4 presents the corresponding captions, offering detailed descriptions for each image type. This combination of visual and textual data forms the foundation for our experimental analysis, enabling a comprehensive understanding of the dataset’s composition and facilitating the evaluation of model performance across different modalities.

### 3.2. Data Preprocessing

A standardized preprocessing pipeline was established to prepare both the image and text data for model ingestion. For images, the PairDxFusion model employed a comprehensive augmentation strategy: all images were first converted to RGB and then subjected to multiple augmentation techniques, including RandomHorizontalFlip (to randomly flip images horizontally and increase variability), RandomRotation (to simulate a range of plausible orientations), and ColorJitter (to vary brightness, contrast, saturation, and hue, thereby enhancing diversity in color presentation). Images were subsequently normalized using the standard ImageNet mean and standard deviation, ensuring compatibility with the pre-trained ResNet backbone [44]. For CLIP-based models, image inputs were processed using the official CLIP ViT-B/32 preprocessing pipeline developed by OpenAI (San Francisco, CA, U.S) to maintain consistency with the model’s original training.

Text preprocessing was deliberately kept lightweight to strike a balance between normalization and semantic preservation. For both PairDxFusion and PairDxCLIP, each caption was first lowercased to normalize casing and minimize vocabulary sparsity, then tokenized via simple whitespace splitting into individual words. Punctuation was stripped during tokenization so that embeddings were mapped only to meaningful tokens. Importantly, abbreviations and negations (e.g., don’t, can’t, MRI) were retained in their original forms–reflecting common clinical language and leveraging the GloVe embeddings ability to capture their semantics. Stopwords were also preserved to retain contextual signals important in multimodal fusion. For the PairDxFusion model, pre-trained 300-dimensional GloVe word embeddings [45] were used to represent each token, and captions were either truncated or zero-padded to a fixed length of 32 tokens, aligning all sequences for batch processing. For the CLIP-based models, the native CLIP tokenizer, which employs a byte pair encoding (BPE) scheme–was used, with no additional text normalization beyond that performed by the tokenizer. This overall approach ensured consistency and clinical relevance, while optimizing input formats for downstream modeling.

### 3.3. Model 1: Baseline Models

**Figure 3:**
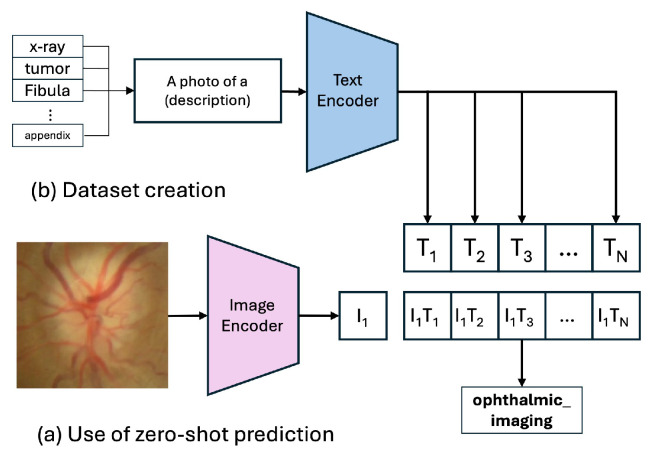
Zero-Shot Prediction of the CLIP Model.

Our baseline model is the pre-trained CLIP ViT-B/32 architecture [7], which we evaluate in a zero-shot setting. This method assesses the model’s inherent capability to generalize to our PairDx dataset without additional training. During inference, the input image and a set of class-descriptive text prompts (e.g., “a photo of a chart”) are encoded using the model’s respective encoders, shown in Figure 3. The resulting feature vectors are L2-normalized [46], which involves scaling each vector to have a unit norm, calculated as in Equation (1):

(1) $$L2-\mathrm{normalized}\;\mathrm{vector}=\frac{v}{{∥v∥}_{2}}$$

where *v* is the feature vector and ${∥v∥}_{2}$ is its L2 norm, defined as $\sqrt{\sum_{i}v_{i}^{2}}$.

Next, the cosine similarity [47] between these vectors is computed, which measures the cosine of the angle between them, as given by Equation (2):

(2) $$\mathrm{Cosine}\;\mathrm{Similarity}=\frac{u\cdot v}{{∥u∥}_{2}{∥v∥}_{2}}$$

where *u* and *v* are the L2-normalized feature vectors. The similarity score is subsequently adjusted by a temperature parameter τ, which regulates the smoothness of the probability distribution. The normalized similarity scores are converted into a probability distribution via the softmax function [48], as seen in Equation (3):

(3) $$\mathrm{Softmax}\left(z_{i}\right)=\frac{e^{z_{i}/\tau }}{\sum_{j}e^{z_{j}/\tau }}$$

where $z_{i}$ are the scaled similarity scores. The class with the highest probability is selected as the prediction. Maintaining this process, the model is tested to predict the class of the input image, without any additional training with our test dataset.

As well, we measure the performance of specialized model—BiomedCLIP, which achieves 66.3% accuracy in the same way.

### 3.4. Model 2: PairDxCLIP (Fine-Tuned Model)

The CLIP model is a robust vision-language model with a dual-encoder architecture, consisting of a Vision Transformer (ViT) for image processing and a Transformer-based text encoder for text input. In our fine-tuning procedure, we fully unfroze both the image and text encoders–in other words, all parameters of the CLIP model were updated, rather than only the classification head or selective layers. No additional adapters or parameter-efficient modules were inserted. To stabilize training, we employed gradient clipping with a maximum norm of 1.0, but did not use learning rate warmup or scheduler techniques. This approach enabled the model to comprehensively adapt its representations to our specific medical image-text domain.

During the fine-tuning process, the pre-trained CLIP model is adapted to the specific domain of medical image-text data. This involves updating the model’s weights using the PairDx training set, which contains domain-specific image-caption pairs. The fine-tuning procedure aims to refine the model’s understanding of medical concepts and improve its classification performance on the PairDx dataset.

By fine-tuning the CLIP model on the PairDx dataset, the model’s representations are shifted from general web-scale concepts to the nuanced features of medical imagery and terminology, resulting in improved classification accuracy.

**Figure 4:**
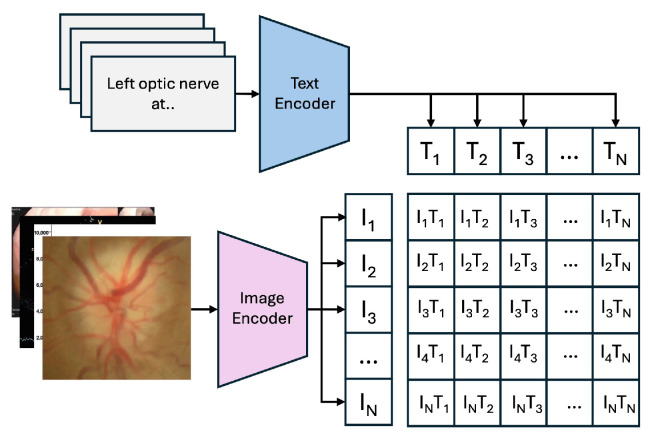
Training Process of the CLIP Model.

Figure 4 illustrates the “Training Process of the CLIP Model,” where the model learns to align images and text in a shared embedding space using contrastive learning. Figure 3 shows the “Zero-Shot Prediction of the CLIP Model,” highlighting its ability to predict image classes by encoding images and text prompts into feature vectors and computing their cosine similarity, without additional dataset-specific training.

The PairDxCLIP model was trained for 10 epochs with a batch size of 128, using the AdamW optimizer with a learning rate of $1\times 10^{-5}$ to minimize the Cross-Entropy Loss. Specifically, we fine-tuned the ViT-B/32 architecture of the general-purpose CLIP model on the PairDx training set, preserving the fundamental structure: a Vision Transformer (ViT) as the image encoder and a masked self-attention Transformer as the text encoder. The goal was to adjust the model’s weights using domain-specific medical image-text data, thereby shifting learned representations from generic web-scale concepts to the nuanced features of medical imagery and vocabulary.

In our custom-built PairDxFusion model, we incorporated a self-attention mechanism in the text encoder branch to dynamically re-weight word embeddings according to their contextual importance within each caption sequence. The attention computation can be mathematically described as follows:

(4) $$\mathrm{Attention}\left(Q,K,V\right)=\mathrm{softmax}\left(\frac{QK^{T}}{\sqrt{d_{k}}}\right)V$$

where *Q*, *K*, and *V* are the matrices of queries, keys, and values, respectively, and $d_{k}$ is the dimension of the key vectors. In our setting, the embedded text sequence $X\in R^{L\times d}$, where *L* is sequence length and *d* is embedding dimension, is linearly projected to obtain *Q*, *K*, and *V*. The resulting weighted sum emphasizes the most salient words, producing a context-aware representation for each caption.

### 3.5. Model 3: PairDxFusion (Custom Multimodal Model)

As a custom-built baseline, we developed PairDxFusion, a multimodal deep learning model featuring a late-fusion architecture. This model uses separate encoders for each modality before fusing their representations. Image Encoder: A ResNet-18 model, pre-trained on ImageNet [49], serves as the image feature extractor. We use the output of the final convolutional block, yielding a 512-dimensional feature vector per image. Text Encoder with Attention: The text branch processes the 300-dimensional GloVe embeddings of the captions. To capture the most salient words, we integrated a self-attention mechanism [50]. This allows the model to dynamically weigh the importance of each word vector in the sequence, producing a context-aware 256-dimensional text representation. Fusion Mechanism: The image and text feature vectors are concatenated and fed into a Fusion Module, which is a Multi-Layer Perceptron (MLP). This module consists of two fully connected layers with ReLU activations and regularization (BatchNorm1d, Dropout) for final classification.

For PairDxFusion, the training was conducted using the AdamW optimizer with a learning rate of 0.001, weight decay of 0.01, a batch size of 128, and a total of 20 epochs, coupled with a OneCycleLR learning-rate schedule. These hyperparameters were chosen to balance convergence speed, generalization, and stability: AdamW was selected for its ability to decouple weight decay from gradient updates, which improves regularization in multimodal models; the learning rate of 0.001 is a widely validated starting point for deep architectures, while OneCycleLR dynamically adjusts it to accelerate early learning and stabilize later epochs; weight decay at 0.01 prevents overfitting by penalizing large weights; a batch size of 128 ensures efficient GPU utilization without exhausting memory; and 20 epochs provide sufficient training iterations to capture multimodal feature interactions while avoiding overtraining. Together, these settings reflect best practices in multimodal deep learning, tuned to achieve robust performance across image–text fusion tasks.

### 3.6. Implementation Details and Experimental Setup

All experiments were conducted on an NVIDIA A100 GPU (40 GB VRAM) within a high-performance computing cluster. The software environment comprised Python 3.10 and PyTorch 2.4.1. Fine-tuning the PairDxCLIP model required 65 min and 52 s, while training the PairDxFusion model required 17 min and 55 s. For testing, both models were evaluated on the held-out test set using the CPU of a MacBook Air M2 (Apple Silicon), also running Python 3.10 and PyTorch 2.4.1. Inference took approximately 0.226 s/image and 0.188 s/image for baseline BiomedCLIP and our own PairDxFusion, respectively. To ensure a fair and direct comparison, both models were trained and evaluated on the identical split under standardized conditions.

## 4. Results and Analysis

This section presents the empirical results of our investigation, beginning with a baseline evaluation of the zero-shot CLIP model, followed by a direct comparison of the fine-tuned PairDxCLIP model and the custom PairDxFusion architecture. All models were evaluated on the same held-out test split of the PairDx dataset to ensure a fair comparison.

### 4.1. Baseline Models Performance

To establish a performance baseline and quantify the “domain gap”, we first evaluated the pre-trained CLIP ViT-B/32 model in a zero-shot setting. As shown in Table 5, the model achieved a modest overall accuracy of 61.18%. While demonstrating a foundational understanding of the classes, its performance was inconsistent across modalities. The model performed exceptionally well on endoscopy (95.30%) and chart (88.15%) images, but struggled significantly with more nuanced classes like electrography (15.46%) and radiology (46.74%), highlighting the limitations of the general-purpose model on a specialized dataset.

**Figure 5:**
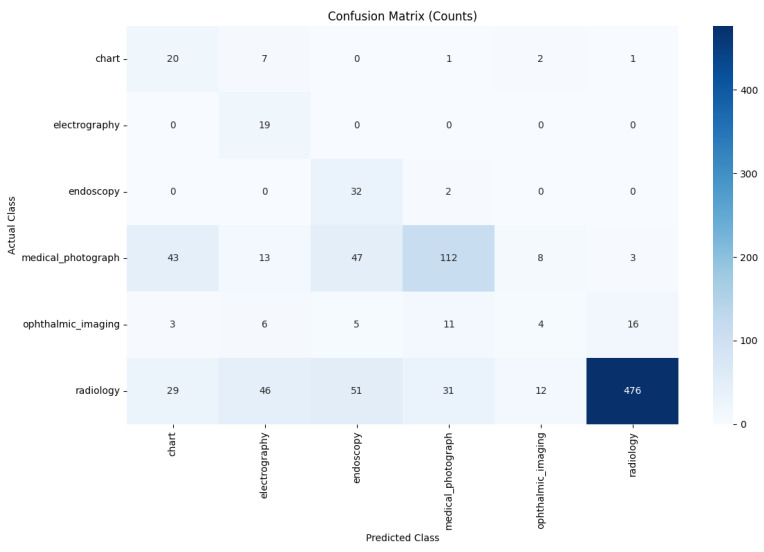
Performance of the BiomedCLIP Model.

Also, we measure the performance of specialized model–BiomedCLIP, which achieves 66.3% accuracy in the same way. The result is shown in Figure 5.

### 4.2. Domain-Adapted Model Performance

**Figure 6:**
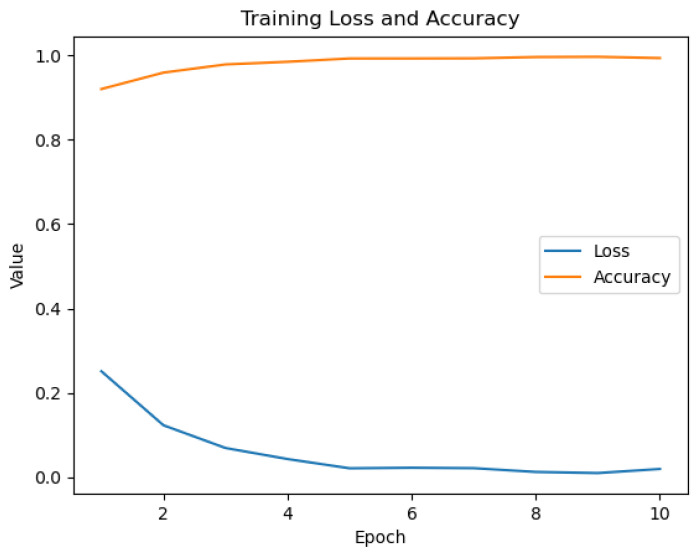
Training history for the PairDxCLIP model over 10 epochs.

The fine-tuning process significantly adapted the general-purpose CLIP model to our specialized medical dataset. The training history (Figure 6) demonstrates rapid and stable learning. Upon evaluation on the unseen test set, the PairDxCLIP model achieved an excellent overall accuracy of 93%, with robust per-class performance (Table 6).

**Figure 7:**
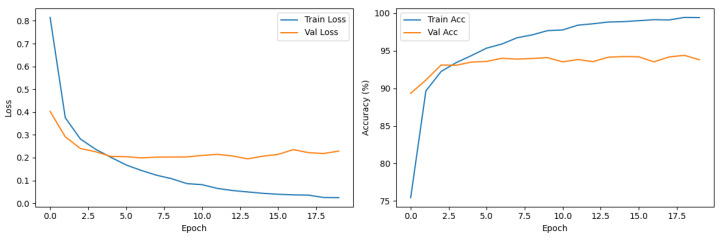
Training and validation loss (**left**) and accuracy (**right**) curves for the PairDxFusion model.

Our custom-built PairDxFusion model also demonstrated strong learning progress, as shown by its training and validation curves in Figure 7. However, the validation accuracy curve plateaus while training accuracy continues to increase, and the validation loss remains consistently higher than the training loss. These patterns are indicative of moderate overfitting: the model fits the training data increasingly well, but does not generalize further to unseen data.

To address such overfitting, several strategies could be considered, including stronger regularization, adding further data augmentation, increasing dropout rates, or employing early stopping based on validation loss. Reducing model complexity or collecting additional data could also help improve generalization.

Despite signs of overfitting, the PairDxFusion model still achieves a high final test accuracy of 94%, matching the fine-tuned CLIP model. This suggests that, while some memorization of training data is occurring, the model is able to capture generalizable patterns sufficient for strong test set performance. The detailed classification report in Table 7 shows consistently high F1-scores across all classes, further supporting the model’s robust generalization within the scope of the current dataset.

### 4.3. Comparative and Qualitative Analysis

To provide a holistic view of the trade-offs between the two approaches, we directly compared their performance and computational efficiency (Table 8). While our custom model achieved an identical test accuracy of 94%, a substantial improvement over the 61.18% baseline, the PairDxFusion model demonstrated a significant advantage in training efficiency, completing its training in just 17 min and 55 s, nearly four times faster than the 65 min and 52 s required for PairDxCLIP. This quantitative evidence supports the hypothesis that a well-designed custom architecture can match the predictive power of a fine-tuned foundation model with substantially lower computational overhead.

**Figure 8:**
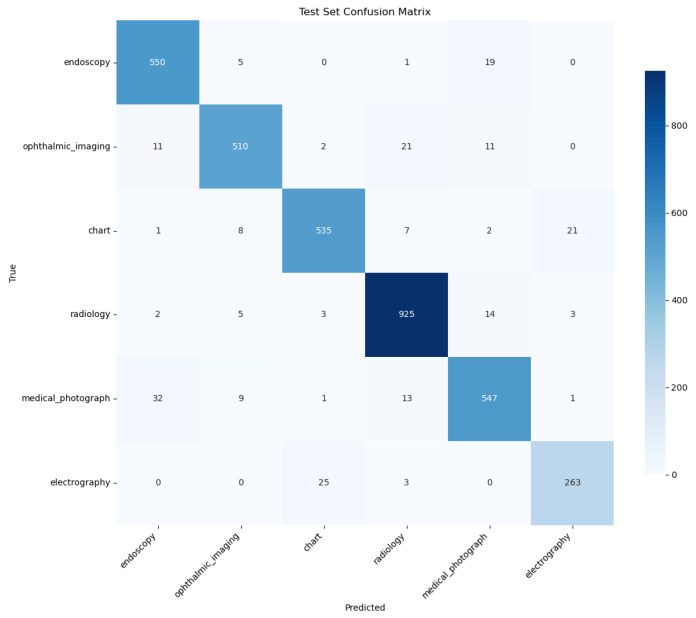
Confusion Matrix for the PairDxCLIP model on the held-out test set.

**Figure 9:**
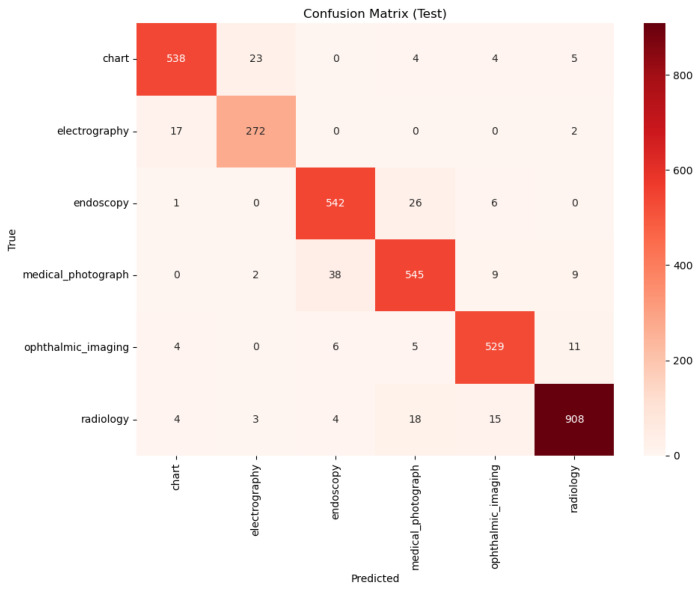
Confusion Matrix for the PairDxFusion model on the held-out test set.

A qualitative comparison of the models’ error patterns using the test set confusion matrices (Figure 8 and Figure 9) reveals distinct predictive behaviors. Both models exhibit strong diagonals, confirming their high overall performance. The PairDxCLIP model shows its most significant confusion between medical_photograph (32 instances) and endoscopy, likely due to visual similarities. The PairDxFusion model shows a similar, though slightly higher, confusion between these two classes (38 instances) but also exhibits minor confusion between radiology and medical_photograph. Both models demonstrate an excellent ability to distinguish the other classes with very few errors.

## 5. Discussion

This study’s findings reveal not just large differences in aggregate accuracy, but also characteristic failure modes for advanced multimodal models in medical document classification. We examined individual samples where PairDxCLIP and PairDxFusion produced different predictions and found that disagreements often centered on ambiguities between medical_photograph and endoscopy. For example, an intraoperative photo with visible surgical tools and a caption referencing surgical resection was correctly classified by PairDxFusion, which leveraged the textual cues, but misclassified as endoscopy by PairDxCLIP, likely due to its reliance on low-level color and texture cues from its foundation model pretraining.

Conversely, in the case of visually ambiguous, sparsely annotated endoscopy images (e.g., a blurry gastrointestinal scene with a generic caption), PairDxCLIP sometimes outperformed the fusion model, benefiting from large-scale visual pretraining, while PairDxFusion failed when the caption provided little context. These case studies suggest that PairDxFusion excels when captions offer clarifying details, whereas PairDxCLIP relies more heavily on visual patterns. Understanding these distinct failure points informs future improvements, such as better caption engineering and enhanced cross-modal fusion strategies.

The results present a clear hierarchy: while the zero-shot general CLIP baseline lags far behind at 61.18%, domain-adapted BiomedCLIP raises the bar substantially to 66.3%. However, both advanced approaches demonstrate state-of-the-art performance, with PairDxCLIP achieving 93% and PairDxFusion slightly surpassing it at 94%, representing a transformative leap over prior baselines. This performance gap is especially noteworthy as it challenges the assumption that only large, fine-tuned foundation models achieve the best results; our custom-designed, from-scratch hybrid architecture not only matches but improves upon their predictive power.

Efficiency further differentiates the models. Table 8 shows that domain-specific BiomedCLIP yields a testing time of 0.226 s per image, whereas PairDxFusion is markedly faster at just 0.188 s per image, a substantial reduction. In terms of overall training efficiency, PairDxFusion completes training nearly four times faster than the foundation model fine-tuning approach. This efficiency gain is crucial for institutions with limited computational resources, as it enables rapid iteration and deployment of high-performing models without access to extensive infrastructure. Thus, practitioners can now strategically weigh the simplicity and potential transfer learning benefits of fine-tuning, the improved but still limited gains from domain-specific models like BiomedCLIP, and the superior efficiency of a tailored hybrid approach, with the knowledge that predictive accuracy need not be sacrificed—and can in fact be enhanced.

The foremost strength of this work is its rigorous, apples-to-apples comparison on our novel, class-balanced PairDx dataset, with explicit quantification of efficiency and accuracy across all baselines and advanced approaches. Nevertheless, limitations persist: our study remains constrained to six modalities, and our custom fusion model uses a straightforward late-fusion mechanism. Furthermore, our evaluation prioritizes predictive accuracy while leaving essential axes such as model interpretability and robustness (especially to out-of-distribution examples) for future investigation.

## 6. Conclusions and Future Directions

In summary, our results show that while domain-specialized models like BiomedCLIP improve upon general-purpose baselines, they still fall short of optimal performance on complex diagnostic tasks. Both a fine-tuned foundation model (PairDxCLIP, 93%) and our custom hybrid model (PairDxFusion, 94%) attained high accuracy, but the custom model offered a slight improvement in accuracy as well as substantial gains in training efficiency.

Our findings highlight that custom hybrid architectures can combine strong performance with lower computational demands, which is especially valuable for practical deployment in clinical settings.

For future work, exploring more advanced fusion strategies (e.g., Transformer-based attention), integrating additional data modalities such as structured EHR, and assessing model robustness and interpretability will help move toward truly reliable and clinically useful multimodal AI systems.

## Figures and Tables

**Table 1:** Comparison of Key Multimodal Medical AI Studies.

Study	Dataset/ Participants	Modality	Model/ Intervention	Main Outcome(s)	Key Limitations
Ye et al. (2024) [38]	MIMIC-III (Retrospective EHRs; ~40,000 adults)	Structured data, Free-text	Hybrid Fusion Neural Net	Acc. 75% (vs. 50% unimodal)	Only structured + text, no images; single-center
Zhan et al. (2024) [20]	MedMNIST, MedNLI (Thousands of cases)	Images, Text	Graph-based Multi-Modal GCN	ROC-AUC up to 0.95	Lacks external validation, mostly imaging
Zhang et al. (2023) [39]	PMC-15M (15 M pairs)	Images, Captions	BiomedCLIP (pretrained)	RadVQA accuracy 80%	Data quality varies, no clinical deployment
Lai et al. (2023) [9]	PCam, BACH, CRC (Public datasets)	Images, Patch Text	CLIPath (PEFT on CLIP)	+19% accuracy on PCam (∼75%)	Short adaptation duration, limited text
Li et al. (2025) [40]	MIMIC (EHR + Images; mixed ages)	Images, Text, Time-series	MDFormer (Transformer)	AUC 85% even with 50% missing data	No real-time test, generalizability unclear

**Table 2:** Performance and Features of Recent Multimodal Fusion Models for Medical Diagnosis.

Study	Model/Framework	Dataset(s)	Key Results	Key Limitations
Li et al. (2025) [40]	MDFormer	MIMIC	Precision 0.8; AUC 85% (robust to missing data)	No prospective validation
Li et al. (2024) [42]	CausalCLIPSeg	QaTa-COV19	Dice: 85.2% (outperforms LViT)	Task-specific, not generalizable
Karimian et al. (2025) [6]	CLIP-IT	PCAM, BACH	+2.87% accuracy (active fusion)	Dependency on text pairing
Chen et al. (2025) [10]	MedCLIP-based	IU-Xray	ROUGE-L: +0.7%	Small gain, no full-stack eval

**Table 3:** Number of Image-Text Pairs in the PairDx Dataset by Modality.

Modality	Number of Pairs
Medical Photograph	4024
Endoscopy	3830
Electrography	1941
Ophthalmic Imaging	3695
Radiology	6345
Chart	3830
Total	22,665

**Table 4:** Image Captions by Label and Type.

Label	Image Type	Image Caption
A	Chart	The levels of carcinoembryonic antigen (CEA) in the cerebrospinal fluid (CSF) and peripheral blood were markedly reduced.
B	Endoscopy	Representative pictures of colonoscopy showing patchy punctate erosion of the terminal ileum. Descending colon.
C	Radiology	Changes in plasma BNP levels after VATS drainage of pericardial effusion and the creation of a pericardial window.
D	Ophthalmic Imaging	Left optic nerve at presentation, showing diffuse swelling.
E	Electrography	Electrocardiogram readings at emergency department. Right electrocardiogram.
F	Medical Photograph	Infection, and dehiscence.

**Table 5:** Zero-Shot Performance of the Baseline CLIP Model on the Test Set.

Class	Accuracy (%)	Correct	Total
chart	88.15%	506	574
electrography	15.46%	45	291
endoscopy	95.30%	548	575
medical_photograph	34.83%	210	603
ophthalmic_imaging	75.32%	418	555
radiology	46.74%	445	952
Average	61.18%	2172	3550

**Table 6:** Classification Performance of the PairDxCLIP Model on the Test Set.

Class	Precision	Recall	F1-Score	Support
chart	0.95	0.93	0.94	574
electrography	0.91	0.90	0.91	291
endoscopy	0.92	0.96	0.94	575
medical_photograph	0.92	0.91	0.91	603
ophthalmic_imaging	0.95	0.92	0.93	555
radiology	0.95	0.97	0.96	952
Accuracy			0.93%	3550

**Table 7:** Classification Performance of the PairDxFusion Model on the Test Set.

Class	Precision	Recall	F1-Score	Support
chart	0.95	0.94	0.95	574
electrography	0.91	0.93	0.92	291
endoscopy	0.92	0.94	0.93	575
medical_photograph	0.91	0.90	0.91	603
ophthalmic_imaging	0.94	0.95	0.95	555
radiology	0.97	0.95	0.96	952
Accuracy			0.94%	3550

**Table 8:** Comparative Summary of Model Performance and Efficiency.

Metric	BiomedCLIP (Specialized Baseline)	PairDxFusion (Custom Model)
Test Accuracy	66.3%	94%
Testing Time	0.226 s/image	0.188 s/image

## Data Availability

The original data presented in the study are openly available at https://zenodo.org/records/17452236 (access on 10 November 2025).

## Abbreviations

The following abbreviations are used in this manuscript:

CLIP	Contrastive Language-Image Pre-training
AI	Artificial Intelligence
GPU	Graphics Processing Unit
EHR	Electronic Health Records
ViT-B/32	Vision Transformer Base with 32 × 32 patches
ResNet-18	Residual Network with 18 layers
GloVe	Global Vectors for Word Representation
MLP	Multi-Layer Perceptron
ReLU	Rectified Linear Unit
BatchNorm1d	Batch Normalization 1D
PEFT	Parameter Efficient Fine-Tuning
CNN	Convolutional Neural Network
